# Metastatic papillary thyroid carcinoma in pleural effusion: a case report and review of the literature

**DOI:** 10.1186/s13256-023-04265-6

**Published:** 2023-12-20

**Authors:** Mohammed Ali Abutalib, Anwar Shams, Shadi Tamur, Eman A. Khalifa, Ghaliah Obaid Alnefaie, Yousef M. Hawsawi

**Affiliations:** 1 Clinical Cytologist and Supervisor of Pathology, Department of Laboratory Medicine and Pathology, Division of Anatomical Pathology, King Abdulaziz Medical City, P.O.Box 9515, 21423 Jeddah, Saudi Arabia; 2 King Abdullah International Medical and Research Center, Jeddah, Saudi Arabia; 3 Department of Pharmacology, College of Medicine, Taif University, P.O. Box 11099, Taif, 21944 Saudi Arabia; 4 Centre of Biomedical Sciences Research (CBSR), Deanship of Scientific Research, Taif University, Taif, 21974 Saudi Arabia; 5 High Altitude Research Center, Taif University, P.O. Box 11099, Taif, 21944 Saudi Arabia; 6 Department of Pediatric, College of Medicine, Taif University, P.O. Box 11099, 21944 Taif, Saudi Arabia; 7 Department of Parasitology, College of Medicine, Taif University, P.O. Box 11099, 21944 Taif, Saudi Arabia; 8 Department of Parasitology, Tanta University, Tanta, Egypt; 9 Department of Pathology, College of Medicine, Taif University, P.O. Box 11099, 21944 Taif, Saudi Arabia; 10 Research Center, King Faisal Specialist Hospital and Research Center, P.O. Box 40047, Jeddah, 21499 Kingdom of Saudi Arabia; 11 College of Medicine, Al-Faisal University, P.O. Box 50927, 11533 Riyadh, Saudi Arabia

**Keywords:** Thyroid cancer, Papillary thyroid carcinoma, Malignant pleural effusion, Aspiration cytology

## Abstract

**Introduction:**

Papillary thyroid carcinoma accounts for the most common type of thyroid cancer of well-differentiated type. Papillary thyroid carcinoma is featured by biologically low-grade and less aggressive tumors with a survival rate of 10 years in most of the diagnosed cases. Papillary thyroid carcinoma can be presented with the involvement of cervical lymph nodes in about 50% of the patients, yet distant spread is very uncommon.

**Case presentation:**

Herein, we discuss a Saudi male patient in his early 50s with a history of papillary thyroid carcinoma who presented to the emergency department complaining of shortness of breath and a radiological finding of hydrothorax. Cytologic examination together with immune-histochemical staining and molecular studies of pleural effusion aspiration concluded the definitive diagnosis of metastatic papillary thyroid carcinoma in the pleural space.

**Conclusions:**

Papillary thyroid carcinoma seldom causes metastatic niches in the pleural space; this is a rare clinical presentation, nevertheless, a differential diagnosis of thyroid metastasis needs to be excluded. A definitive diagnosis of metastatic papillary thyroid carcinoma can be made using clinical presentation, cytologic examination, immunohistochemical investigation, and molecular testing. The most common mutation found in papillary thyroid carcinoma cases is the V600E mutation found in the *BRAF* gene, yet these patients have a relatively low probability of cancer recurrence. Patients with papillary thyroid carcinoma who have the *BRAF* mutation frequently experience metastases and relapses of the disease after the cancer has progressed aggressively. To help with therapy planning and the introduction of* BRAF* inhibitors, genetic testing for *BRAF* mutation may therefore prove to be a useful tool, especially in cases of aggressive subtypes of TC.

## Introduction

Thyroid cancer (TC) is the most common endocrine malignancy [1]; TC showed a global prevalence of 586,202 cases and an estimated 43,646 deaths in 2020 [2]. Until recently, TC displayed a highly growing incidence in the USA [3]; the American Cancer Society estimates 43,800 adults (11,860 men and 31,940 women) in the USA will be diagnosed with TC in 2022 [2]. However, from 2014 to 2018, about a 2.5% annual drop in the incidence rate of thyroid cancer was observed, which was most likely due to improvement in the healthcare system and the avoidance of overdiagnosis [2, 4, 5]. On the other hand, the mortality rate of thyroid cancer cases remained stable from 2010 to 2019, with an estimated 2230 (1070 men and 1160 women) death cases from TC in the USA happening this year [2]. TC is considered the seventh most common cancer among women (31,940 cases) in 2022 [2] and showed a women-to-men ratio of 3:1 [6, 7]. Indeed, disparities in cancer incidence, prognosis, and aggressiveness have been noted among various cancer types in both genders, including thyroid carcinomas. Less aggressive histopathological subtypes are typically associated with female TC, whereas the aggressive phenotype was found to be equally distributed in both sexes [8]. This suggests that the occurrence of TC in men is mostly followed by an advanced course of the disease and is sometimes accompanied by distant metastases at the initial presentation [9]. A retrospective study was conducted to improve the risk stratification system of the American Thyroid Association (ATA). This study analyzed 1547 papillary thyroid cancer (PTC) cases (1358 females and 189 males) from 1986 to 2018 and revealed that male gender can be considered as a crucial risk factor for predicting poor clinical course. They reported that men with PTC, as compared with women, presented with a progressive disease, advanced pathological features, blood vessels and lymph node metastasis, tumor size over 40 mm, and unfavorable responses to the initial therapy [10].

Among the Saudi population in the central region of Riyadh, TC was reported to be the second most common occurrence of cancer between the 2000 to 2010 period, which was predominant in female cases as compared with male cases (1:0.3). Over the past years, an increasing incidence of thyroid cancer has been reported in Saudi Arabia. However, the etiology of thyroid cancer is still not clear. A study was conducted in Saudi Arabia from 1990 to 2019 to estimate TC incidence and mortality using Global Burden of Disease (GBD) web-based tools. This report showed that the total number of diagnosed TC is 23,846 cases (17,220 females and 6626 males), predominantly in females. The TC incidence progressively increased by 15-fold among females and by 22-fold among males. Additionally, the death rate steadily increased by threefold in women and interestingly by sixfold in men in the same period. The author attributed the increase in the incidence rate to the advancement in the tools of detection and diagnosis [11]. Indeed; the most detected subtypes of TC were papillary adenocarcinomas, mixed papillary and follicular carcinoma, papillary microcarcinoma, papillary columnar cell carcinoma, and papillary carcinoma encapsulated subtypes, respectively [12]. Moreover, a systematic meta-analysis study was conducted to determine the incidence of different types of cancers in Saudi Arabia through the era of 2010 to 2019 [13]. This study revealed that the TC prevalence rate accounts for 12.9% of the diagnosed and reported cases in the Saudi population. Certainly, TC occupied the third-ranking type of cancer in the Saudi population among both genders with a 10.1% incidence rate and a 1.4% mortality rate [13–16]. Additionally, TC was found to be the second most common cancer among Saudi women after breast cancer, according to Saudi Cancer Registry (SCR) [16, 17]. Previous family history of thyroid cancer and goiter, presence of benign thyroid condition and certain genetic diseases, radiation exposure, low iodine intake, increased body weight with elevated leptin level, and female gender were identified as the major risk factors for the development of thyroid cancer [18–21]. In addition to these risk factors encountered by the Saudi population, increased screening using sensitive tools such as ultrasonography could be attributed to the growing incidence of TC in the Saudi community [13, 15, 22, 23].

Based on pathological and histological appearance, TC can be classified into a follicular adenoma, hyalinising trabecular tumor, encapsulated follicular-patterned thyroid tumors, papillary thyroid carcinoma (PTC), follicular thyroid carcinoma, Hürthle cell tumor, poorly differentiated thyroid carcinoma, anaplastic thyroid carcinoma, and squamous cell carcinoma. These tumors can be further categorized into benign, malignant, or borderline [18]. PTC is the most common type of thyroid cancer (~ 80%) and has more occurrence among females compared with males, which is usually diagnosed in the third to fifth decade of patients’ age [19, 24]. The follicular cells of a healthy thyroid gland are the source of PTC. Tumor cells grow in a papillary pattern, exhibiting three unique nuclear features: (1) enlarged and overlapping nuclei, (2) pale and optically clear nuclei, and (3) irregularities in the nuclear membrane [5]. Numerous PTC variations have been discovered, each with a distinct prognosis and course of events [25]. PTC is characterized by less aggressive behaviors and good patient prognosis [19, 24]. With a 93% survival rate at 10 years, PTC is one of the most treatable cancers worldwide when compared with other differentiated and undifferentiated thyroid malignancies [26]. PTC metastasizes to local lymph nodes in between 30 and 40% of cases [27]. However, 1–4% of patients may develop distant metastases, which lowers the survival rates to 24–76% [28]. The most typical locations for distant metastases are the lungs and the bones. Remarkably, the 5-year survival rate for patients with multiorgan metastasis is 15.3%, a sharp drop from the 77.6% rate for patients with single-organ metastasis [25, 29]. Distant metastases of PTC are uncommon, and metastatic pleural effusion as the initial presentation in PTC is very rare [24, 30, 31], though few cases have been previously reported [31–33].

Often, the first diagnostic technique used to find PTC is fine needle aspiration. Typically cellular, fine-needle aspiration specimens from conventional PTC may display papillary structures, monolayer sheets, and three-dimensional (3D) groups against a background of nuclear or calcific debris, macrophages, and stromal fragments, as well as thick or watery material known as “ropy colloid.” PTCs are radiographically typically seen as cold (hypofunctioning) nodules, though they can also occasionally be seen as hot (hyperfunctioning) nodules. For PTC, ultrasound examination is the preferred imaging modality. The results of ultrasounds are also very helpful in directing fine-needle aspiration biopsies of aberrant nodes. This kind of cancer is highly specific to microcalcifications. To determine the degree of extrathyroidal extension, determine whether substernal masses are present, find recurrent tumors, and enhance diagnostic precision, additional imaging modalities, such as computed tomography (CT), magnetic resonance imagine (MRI), and fluorodeoxyglucose (FDG)-positron emission tomography (PET)/CT, might be required [29]. The clinical history, cytologic examination, immunohistochemistry study, and molecular testing are crucial to accurately diagnose metastatic papillary thyroid carcinoma in the pleural space [34].

In the current report, we describe an extremely rare presentation of PTC that metastasized in the pleural space in a male patient in his early 50s. This patient with a metastatic PTC presented the only incidence case that was diagnosed in King Abdulaziz Medical City in Jeddah, Saudi Arabia between 1999 and 2020. A definitive diagnosis of metastatic PTC into pleural effusion was achieved using different diagnostic tools including imaging and cytopathology analysis. Furthermore, genetic testing of the original tumors revealed a positive mutation of the *BRAF*^V600E^ gene, thus justifying the aggressive manner of PTC in the current study. To the best of our knowledge, this is the first study to report an occurrence of a metastatic PTC presenting with malignant pleural effusion in the Kingdom of Saudi Arabia.

## Case presentation

**Fig. 1:**
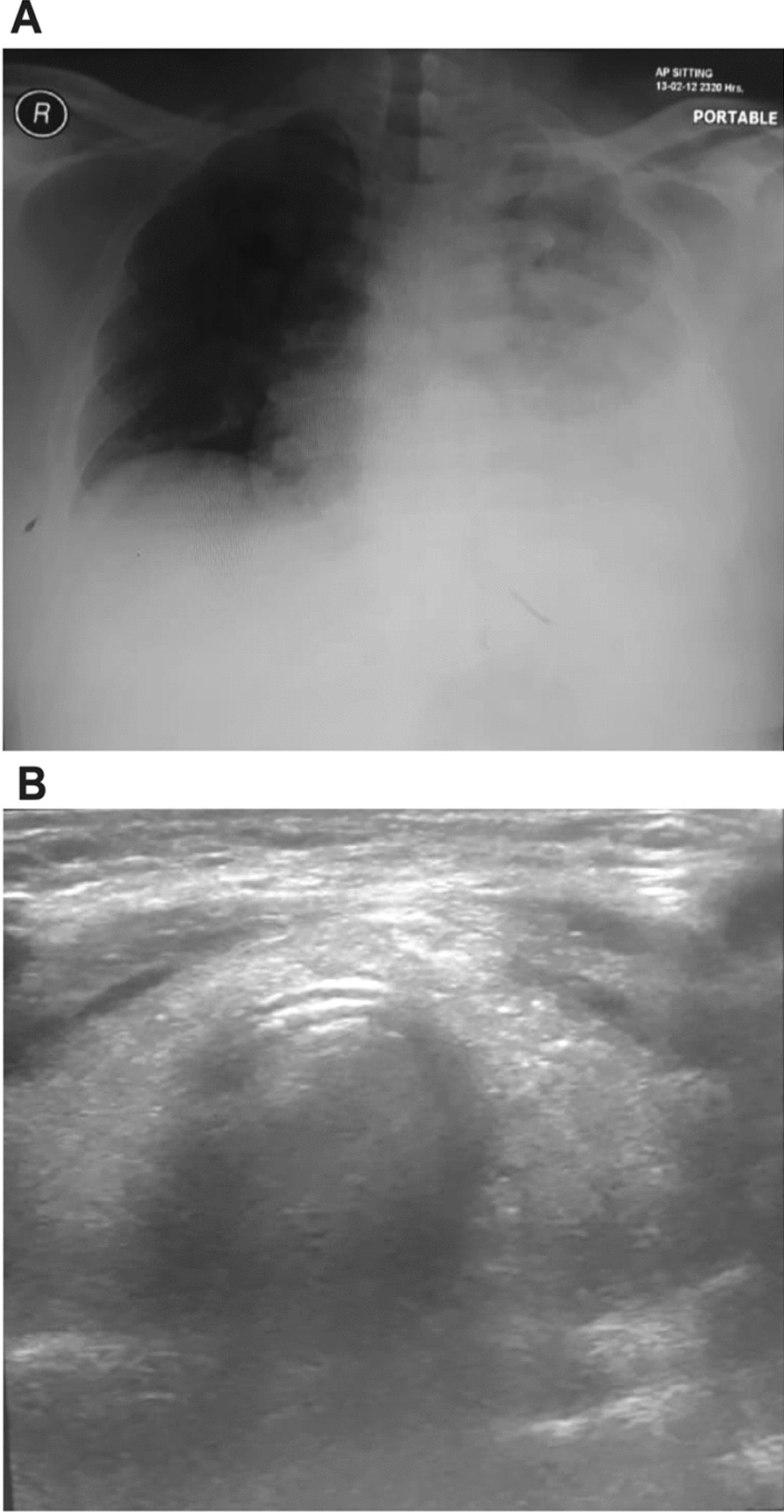
X-ray and ultrasonography of the chest: chest X-ray showing left side pleural effusion with mediastinal shifting to the right, the right lung is clear (**A**).**B** Ultrasonography of the lungs shows a left-sided collection of pleural effusion presenting as a crescent-shaped hypoechoic area measured as 8 × 1.9 cm

A Saudi male in his 50s, chronic smoker, with a past medical history of papillary thyroid carcinoma presented to the emergency department complaining of severe shortness of breath and chest pain. The past medical and surgical history also demonstrated total thyroidectomy and an administration of the LENVIMA chemotherapy on a daily basis for 4 weeks. In the emergency department, chest radiology showed left-side hemithorax opacity associated with mediastinal shifting to the right side. This finding is consistent with a large left-side pleural effusion, while the right lung is clear, as shown in Fig. 1: X-ray and ultrasonography of the chest (Fig. 1A). Ultrasound of the chest showed a left-sided collection of pleural effusion presenting as a crescent-shaped hypoechoic area measured as 8 × 1.9 cm (Fig. 1B). Radiology examination provides a high specific yet less sensitive diagnostic tool for the detection of pleural malignancy [35]. To achieve the conclusive diagnosis, the patient underwent thoracentesis, a procedure to obtain fluid from the space surrounding the lungs, and around 150 ml of bloody fluid was aspirated from the pleural space for biochemical analysis and cytopathologic evaluation. Cytologic examination revealed the presence of an exudative effusion.

**Fig. 2:**
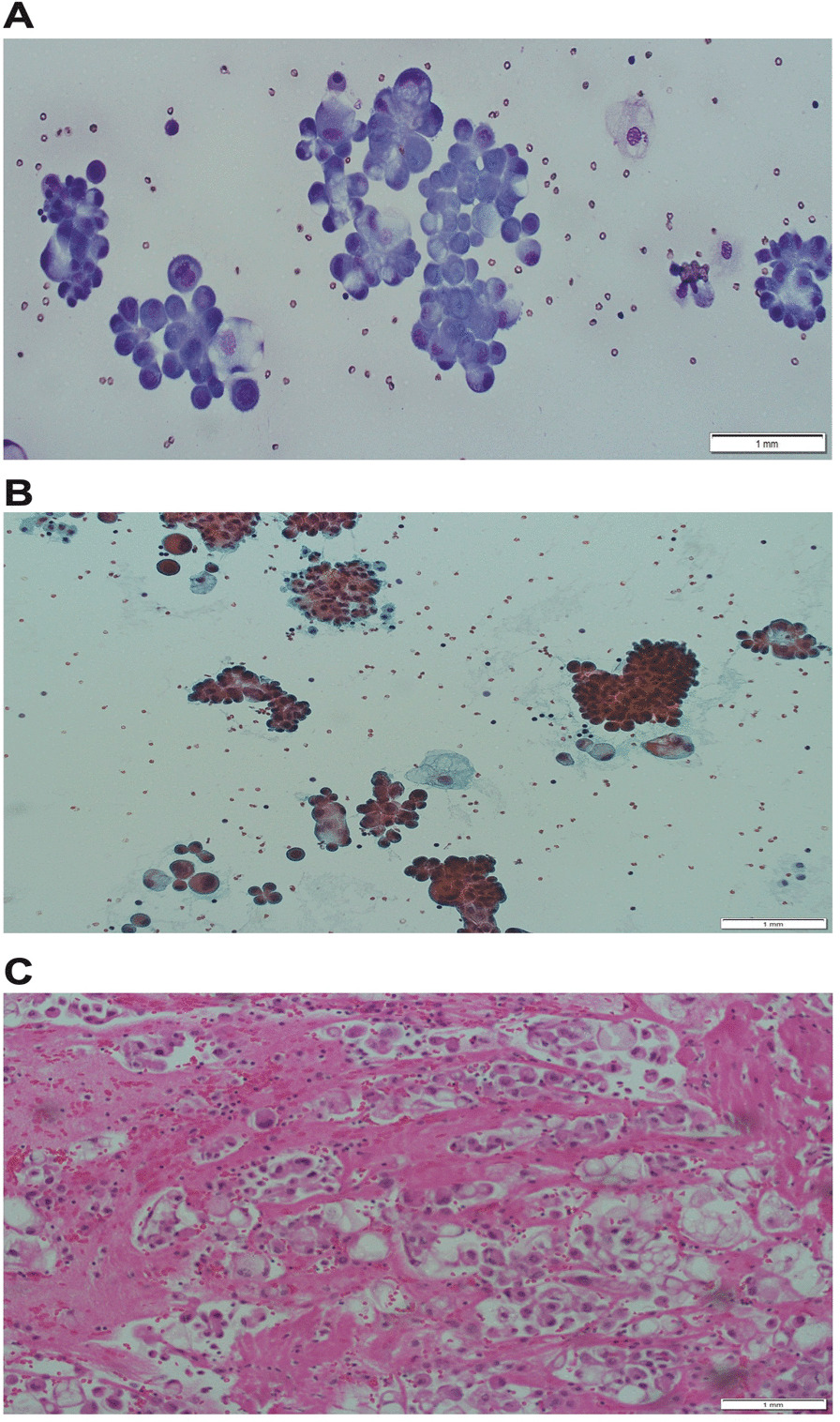
Cytopathology analysis of pleural effusion aspiration: two slides using the cytospin technique were prepared, one slide was stained by Diff Quick (**A**) and the other was stained by Papanicolaou stain (**B**). Cellblock technique was performed from the sediment after concentration technique (centrifugation), and one slide was stained with hematoxylin and eosin (H&E); stains were very cellular and showed predominant malignant cells in the papillary cluster, pleomorphic nuclei, cytoplasmic vacuolization, intranuclear inclusion, and powdery chromatin in a background of few polymorphous and reactive mesothelial cells (**C**). Magnification in all slides is 20×

As shown in Fig. 2: cytopathology analysis of pleural effusion aspiration (Fig. 2A, B); preparation of the cytology slides was performed using Diff Quick and Papanicolaou stains to aid in making differentiation and diagnosis of the pathological specimen. Hematoxylin and eosin (H&E) staining was also performed and demonstrated the presence of highly proliferative malignant cells in a papillary cluster with pleomorphic nuclei, cytoplasmic vacuolization, and intranuclear inclusion features (Fig. 2C). Immunohistochemistry (IHC) was used to assess the level of various biological markers to assist in determining the final diagnosis of metastatic papillary thyroid carcinoma in pleural effusion.

**Fig. 3:**
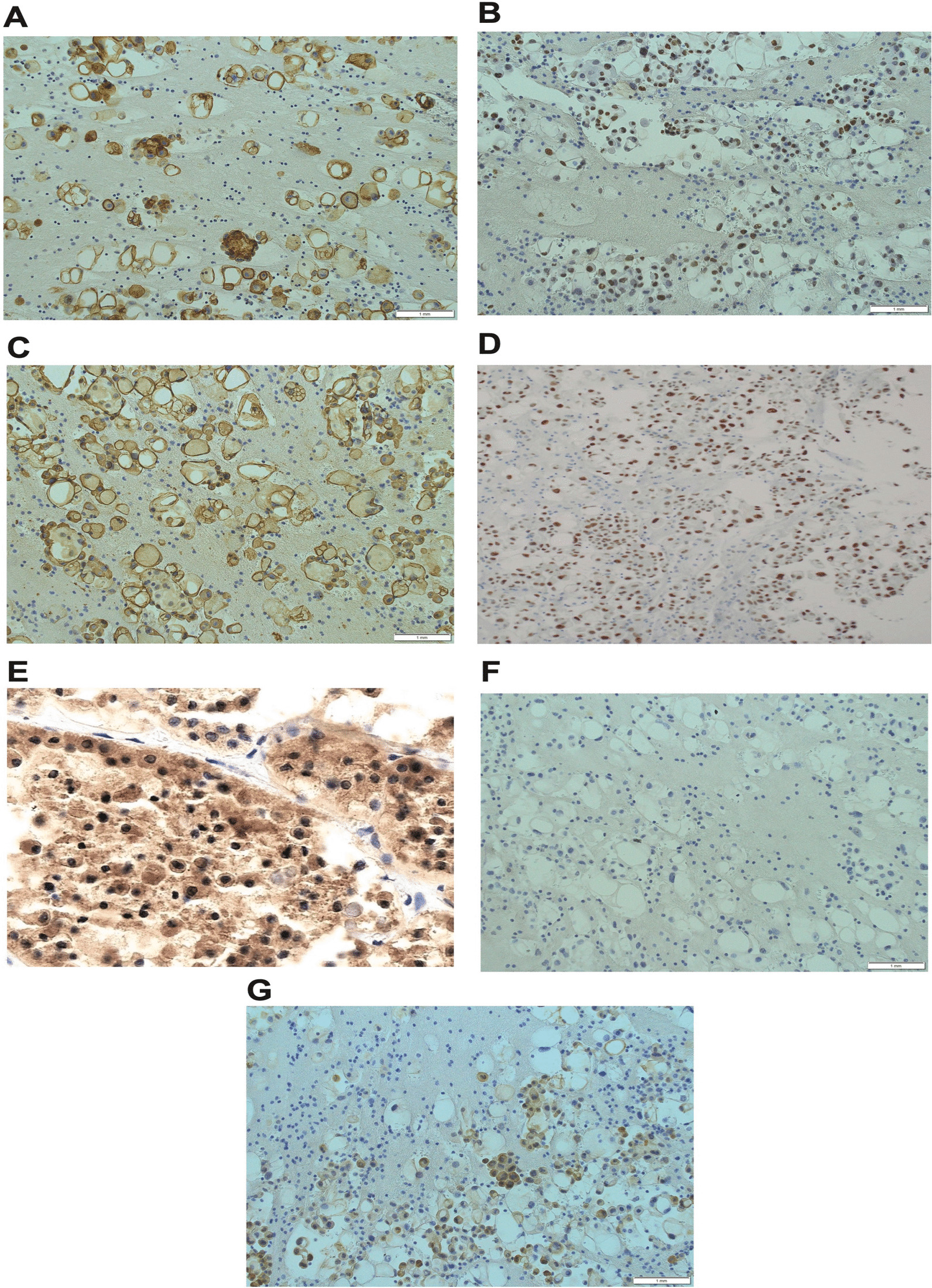
Immunohistochemistry studies for different biological markers: The neoplastic cells showed positive staining for Ber-EP-4 (**A**), thyroid transcription factor-1 (TTF-1) (**B**), CK-7 (**C**), Pax-8 (the confirmatory test) (**D**), and thyroglobulin (**E**). Neoplastic cells showed negative staining for CK-20 (**F**) and clathrin (**G**). Magnification in all slides is 20×

As shown in Fig. 3: immunohistochemistry studies for different biological markers (Fig. 3A–G); IHC analysis revealed positive staining of different markers that were reported to associate positively with the development of PTC including cytokeratin-7 (CK-7) [36], thyroid transcription factor-1 (TTF-1) [37], thyroglobulin [38], and a thyroid-specific transcription factor; human paired box-8 (Pax-8) was identified as the most dysregulated target gene in PTC [39]. Furthermore, the metastatic tumor cells exhibited positive results when using Ber-EP-4, a monoclonal antibody [40], which was found to be positive in metastatic PTC [41] and is used to differentiate epithelial from mesothelial cells [18]. On the other hand, the cancer cells showed negative IHC results for cytokeratin-20 (CK-20) [36] and clathrin [42]. Thus, confirming the diagnosis of metastatic PTC. In addition, molecular pathology analysis of the original tumor was performed in Germany for the detection of predictable genetic alterations such as *BRAF*^V600E^ and NRAS gene mutations. *BRAF*^V600E^ mutation was positive in the tumor samples while no alteration in the *NRAS* gene was observed.

## Discussion

Papillary thyroid carcinoma is the most common type of low-grade thyroid cancer with favorable patient outcomes. PTC is more predominant among females around the age of 40–50 years [19, 24]. However, when PTC is diagnosed in male patients, it is mostly manifested by aggressive presentations, advanced clinical staging, and postoperative residual disease [9]. In the current case, we present a male patient with a past medical history of PTC manifested by metastatic malignant pleural effusion, a very rare and aggressive consequence of PTC. Among 267 patients who were registered at King Abdulaziz Medical City in Jeddah, Saudi Arabia between 1999 and 2020 with a diagnosis of papillary thyroid cancer, one patient (1/267 = 0.4%) had metastatic papillary thyroid carcinoma. The detection of malignant pleural effusion was made 30 months after the initial diagnosis of PTC. Ten months later, after discovering the metastasis, the patient’s medical condition progressed and he passed away, thus authenticating that such presentation of PTC was found to be correlated with poor prognosis and shortened overall survival [31, 43]. A retrospective report of patients with cancer with metastatic follicular-cell-derived thyroid carcinoma from 1990 to 2010 displayed that involvement of metastatic pleural effusion was identified as a poor and an unfavorable prognostic factor in these patients [44]. The previously reported cases of similar presentation of metastatic pleural effusion, either secondary to primary PTC [31, 32], or anaplastic transformation of a well-differentiated PTC [33] are very limited worldwide and mostly were detected in the Japanese population, which demonstrated a similar aggressive course of the disease terminated by patient death.

Papillary thyroid carcinoma is an indolent disease and distant metastasis is infrequent. When distant spread secondary to residual PTC is detected, it commonly involves cervical lymph nodes, lungs, and bones [32, 45]. About 67% of malignant pleural effusions are secondary to lung cancers, breast cancers, gastrointestinal tract malignancies, and malignant lymphomas followed by ovarian carcinoma [43, 46]. Malignant pleural effusion due to thyroid cancer has been reported to be less than 1% in all cases [43, 47], and this is in alignment with our findings. Comprehensive clinical history and physical examination accompanied by appropriate solid diagnostic tools are essential in approaching an accurate diagnosis and hence providing a better therapeutic plan when applicable. Fine needle aspiration and cytology (FNAC) is considered the gold standard technique in the diagnosis of PTC with a high specificity of 90% [32, 48]. Pleural fluid cytology is the simplest and most definitive method to diagnose the metastatic involvement of the pleural space; however, the diagnostic yield of pleural fluid cytology ranges from 40% to 87% [49].

Four factors are important when considering the primary site of metastatic malignancies, including: the type of cells present in the effusion the location of effusion, the age and sex of the patient, and the nature of the tumor at the distant site [50]. Using different diagnostic strategies including cytology, IHC, and molecular genetic analysis, we were able to render a definitive diagnosis of an unusual and very infrequent case of metastatic papillary thyroid carcinoma in pleural effusion, which showed a marked papillary neoplastic structure characterized by positive expression of specific pathological markers such as CK-7 and TTF-1. A large cohort of TC of a 20-year computerized search of effusion cytology revealed that cytological findings, along with clinical history and immunohistochemical techniques, are useful tools to achieve the correct final diagnosis [51].

Moreover, the molecular genetic test of the tumor specimen indicated the presence of the *BRAF*^V600E^ mutation. *BRAF* activation in TC can result from several genetic abnormalities, including point mutation and chromosomal rearrangement [18]. *BRAF* belongs to the *RAF* family of serine–threonine kinases that play a role in regulating the *RAS*/*MAPK* pathway. This pathway contributes to mediating different cellular functions such as proliferation, differentiation, and apoptosis [18]. Indeed, *BRAF*^V600E^ mutation in patients with PTC was found to be associated with the activation of phosphoinositide 3-kinase-Akt serine/threonine kinase (*PI3K*-*AKT*) pathway, resulting in a more aggressive cancer phenotype [52]. *BRAF*^V600E^ mutation was reported to be the most common genetic abnormality in ~ 90% of all thyroid cancers [18, 53] and was found to be harnessed with older-age patients, lymph nodes and distant metastases, advanced TNM staging, recurrence and progressive disease [54], and poor patient outcomes [55, 56]. Nevertheless, *BRAF*^V600E^ mutation is the most frequent mutation found in conventional PTC (~ 51%); the overall recurrence risk rate is low, ranging from 1% to 6%, and those patients are classified as a low-risk recurrence group [57]. Additionally, a recent comprehensive meta-analysis was conducted in 2020 to reevaluate the association between the *BRAF*^V600E^ mutation and the recurrence of PTC [58]. In this analysis, about 11 studies composed of 4674 patients were reviewed using different online available databases and the HR value as a measurable tool to compare between the patient with a *BRAF* wild type and the mutated type. The results of the study concluded that to build a significant association between the existence of *BRAF* mutation and the risk of PTC relapse, both geographical area and stage of the disease together with clinical presentation should be contemplated [58]. Based on this data, it is not routinely recommended in the clinic to test for *BRAF* status for the initial presentation of differentiated thyroid cancer (DTC) such as PTC, particularly in low-risk patients who do not present with worrisome features. While those patients who displayed multifocal tumors and signs of invasion were stratified as intermediate-risk of recurrence, testing for the *BRAF* mutation could play a role in deciding on a management plan [57].

In either view, testing for this mutation could provide substantial value and potentially assist in decision making for therapeutic and follow-up planning of patients with TC [54]. For example, in another rare and vastly fatal TC, anaplastic thyroid cancer (ATC), the 2021 guidelines by the American Thyroid Association strongly recommended assessment of *BRAF*^V600E^ mutation by IHC and molecular testing after the definitive diagnosis of ATC is documented to draw a clear therapeutic plan [59]. A combination regimen consisting of *BRAF* inhibitors—dabrafenib plus trametinib—was approved by the FDA for ATC-positive *BRAF*^V600E^ mutation and resulted in significant tumor volume regression and 100% control of the locoregional spread [59]. Also, the safety of this combination therapy of dabrafenib plus trametinib for the treatment of *BRAF*^V600E^ mutation-positive patients with ATC was recently reevaluated in a phase II Rare Oncology Agnostic Research (ROAR) basket study. This combination therapy revealed significant clinical benefits, tolerable and controllable toxicity, and prolonged survival periods for the treated group [60]. In patients with PTC harboring positive *BRAF* tumors, the introduction of this combination therapeutic approach has been proposed and several clinical trials have been conducted to investigate this strategy [61].

Besides the *BRAF*^V600E^ mutation, the genetic test of the tumor did not show any mutation in *NRAS*, a member of the *RAS* gene family. The *RAS* gene belongs to the GTP-binding protein family that has an essential role in regulating cellular proliferation through *MAPK* and *PI3K*-*AKT* pathways. The *RAS* gene family consists of three members: *HRAS*, *NRAS*, and *KRAS*. Mutations of these genes have been reported in TC [18]. Mutation in the *NRAS* gene was found to be more commonly harnessed to poorly differentiated thyroid carcinomas and anaplastic thyroid cancers than PTC [62]. Furthermore, subsequent genetic mutational studies have identified the *RET* gene chromosomal rearrangement contribution to the development of PTC. *RET* is a protooncogene that can activate the oncogenic MAPK cascade. *RET* encodes plasma membrane-bound RET tyrosine kinase receptors, and the RET protein is expressed mainly in the parafollicular cells, or C cells, of the thyroid gland. Mutations in the genes *BRAF*, *RAS*, or *RET* constitute around 70% of PTC cases and may result in different variants of PTC such as the classical, follicular, tall cell, and columnar cell variants [34].

## Conclusion

Malignant effusion from papillary thyroid carcinoma is an infrequent and uncommon finding. The clinical presentation, cytologic examination, and immunohistochemistry study in conjunction with molecular testing are valid approaches to reaching the decisive diagnosis of metastatic papillary thyroid carcinoma. *BRAF*^V600E^ mutation accounts for the most frequent mutation detected in PTC cases, yet the cancer recurrence rate is quite low in these patients. Patients with PTC harboring *BRAF* mutation and experiencing a relapse of the disease and metastases mostly follow an aggressive and progressive course of the disease. Therefore, genetic testing for *BRAF* mutation could offer a valuable tool in aiding the decision making for therapeutic planning and introduction of *BRAF* inhibitors, particularly in aggressive subtypes of TC.

## Data Availability

The datasets used and/or analyzed during the current study are available from the corresponding author upon reasonable request.

## Abbreviations

TC: Thyroid cancer
SCR: Saudi Cancer Registry
PTC: Papillary thyroid carcinoma
IHC: Immunohistochemistry
CK-7: Cytokeratin-7
TTF-1: Thyroid transcription factor-1
Pax-8: Human paired box-8
PI3K-AKT: Phosphoinositide 3-kinase-Akt serine/threonine kinase
DTC: Differentiated thyroid cancer
ATC: Anaplastic thyroid cancer
ROAR: Rare Oncology Agnostic Research
